# 6‐Month Clinical Evaluation of Reduced Dentin Etching Time Using Universal Adhesives in Noncarious Cervical Lesions: A Double‐Blind Randomized Clinical Trial

**DOI:** 10.1111/jerd.70224

**Published:** 2026-07-02

**Authors:** Mario Felipe Gutiérrez, Natalia Amo‐Nicolás, Alondra Beniscelli‐Vásquez, Romina Aliaga‐Gálvez, Alessandro D. Loguercio

**Affiliations:** ^1^ Facultad de Odontología Universidad de los Andes Santiago Chile; ^2^ Centro de Investigación e Innovación Biomédica (CIIB) Universidad de los Andes Santiago Chile; ^3^ School of Dentistry State University of Ponta Grossa Ponta Grossa Brazil

**Keywords:** clinical trial, dental etching, dentin‐bonding agents, tooth cervix, tooth wear

## Abstract

**Objectives:**

To evaluate the effect of reduced dentin etching time on the clinical performance of two universal adhesive systems in noncarious cervical lesions (NCCLs).

**Methods:**

A total of 144 NCCLs restorations were randomly placed in 36 participants according to four experimental groups: SUP5 (Scotchbond Universal Plus adhesive [pH ≈2.5–2.7] applied on dentin etched for 5 s [experimental group]); SUP15 (SUP applied on dentin etched for 15 s [control group]); GBU5 (Gluma Bond Universal adhesive [pH ≈1.5–1.8] applied on dentin etched for 5 s [experimental group]); GBU15 (GBU applied on dentin etched for 15 s [control group]). Restorations were evaluated at baseline and after 6 months, using the updated FDI criteria. Appropriate statistical analyses were performed (*α* = 0.05).

**Results:**

The recall rate was 100% at 6 months. Retention/material fracture rates were 97.2% (87.3–100) for SUP5 and SUP15, and 94.4% (83.1–100) for GBU5 and GBU15. No significant differences among the groups were observed for retention or material fracture. Two restorations showed minor marginal staining, two restorations showed minor postoperative (hyper‐) sensitivity, and 25 restorations presented minimal marginal adaptation defects. No significant differences were observed among the groups for any secondary clinical parameters evaluated (*p* > 0.05).

**Conclusion:**

Reducing dentin etching time did not negatively affect the 6‐month clinical performance of NCCLs resin restorations bonded with universal adhesives. Therefore, shorter dentin etching times represent a viable clinical approach in adhesive clinical procedures, reducing chair time.

**Clinical Significance:**

Shortened dentin etching time does not compromise the short‐term clinical performance of universal adhesive systems in NCCLs.

## 1 Introduction

Adhesive dentistry aims both to increase the longevity of tooth‐restoration interfaces and to simplify restorative techniques [1]. To accomplish these objectives, clinicians have increasingly adopted universal adhesives due to their reduced technique sensitivity [2, 3], enhanced operator convenience, and suitability for multiple application modes [4, 5, 6]. The versatility of universal adhesives is largely attributed to the incorporation of acidic functional monomers within methacrylate‐based formulations. These monomers—including 4‐methacryloxyethyl trimellitic acid anhydride (4‐META), glycerol phosphate dimethacrylate (GPDM), and 10‐methacryloyloxydecyl dihydrogen phosphate (10‐MDP)—promote chemical bonding to tooth substrates and metal oxides through ionic interactions with calcium ions or surface hydroxyl groups. Among these, 10‐MDP has been the most extensively investigated due to its long hydrophobic carbon chain, which confers superior hydrolytic stability and strong bonding to hydroxyapatite [7, 8, 9, 10].

Beyond its polymerization potential, 10‐MDP is capable of chemically interacting with dental substrates [3, 11, 12], following the adhesion route of the adhesion‐decalcification (AD) concept. According to this concept, acidic functional monomers can either form hydrolytically stable ionic bonds with calcium ions of hydroxyapatite—the adhesion route—or promote extensive calcium removal from the tooth surface—the decalcification route [13, 14, 15]. Unlike strongly acidic monomers or phosphoric acid, which predominantly follow the decalcification route, 10‐MDP undergoes limited partial demineralization of hydroxyapatite, preserving residual hydroxyapatite crystals at the bonded interface while establishing stable ionic interactions with calcium ions, leading to the formation of organized nanolayers of insoluble 10‐MDP‐calcium salts [16]. Although the extent of nanolayering depends on the final concentration of 10‐MDP applied to dentin [17], the presence of residual hydroxyapatite at the bonded interface is essential to generate efficient chemical interaction. When these interactions are stable, tooth‐restoration interfaces would successfully resist several oral challenges such as mastication loads and hydrolytic degradation.

Universal adhesives are also designed to simplify dentin hybridization procedures and may be applied using etch‐and‐rinse, self‐etch, or selective enamel‐etching application modes [18, 19, 20, 21, 22, 23, 24]. In the etch‐and‐rinse approach, phosphoric acid at concentrations ranging from 32% to 40% promotes complete removal of the smear layer and smear plugs [25], while simultaneously demineralizing intertubular dentin and exposing the organic collagen matrix. This demineralization creates nanoscale porosities within the collagen fibril network [26], thereby facilitating the penetration of adhesive monomers into and around the collagen framework, forming a resin‐reinforced transitional zone, a biologic composite in which resin monomers become interpenetrated with demineralized collagen fibrils and polymerize in situ [27], providing effective micromechanical interlocking at the resin‐dentin interface [6, 28, 29, 30].

However, there are certain challenges related to the acid etching process on dentin, particularly regarding the remnant hydroxyapatite at the bonded interface, the depth of dentin demineralization, and the increased density of exposed dentinal tubules [26, 31]. Excessive demineralization may reduce the availability of calcium for stable ionic bonding, compromise the formation of 10‐MDP–calcium salts, and create discrepancies between the depth of demineralization and adhesive monomer infiltration. These discrepancies can result in areas of exposed collagen beneath the hybrid layer that are incompletely infiltrated [32], serving as pathways for degradation of the resin–dentin bond over time. This degradation process is largely mediated by endogenous dentinal matrix metalloproteinases and cysteine cathepsins [33, 34] that are released and activated by phosphoric acid and acidic monomers in adhesive systems [35].

Despite the fact that most manufacturers recommended a dentin etching time of 15 s [36], recent investigations have demonstrated that this etching time could remove a large portion of the mineral content of the dentin, resulting in pronounced exposure of the collagen matrix. This finding suggests that the conventional etching time may be excessive for achieving an effective dentin etching pattern [26, 31]. In this sense, reducing dentin etching time seems to be a clinically practical strategy [37], as it does not add additional steps into the adhesive protocol and preserves the simplicity and reduced technique sensitivity that characterize universal adhesives. In vitro studies have demonstrated that shorter dentin etching time (“short dentin etching” [38] or “selective dentin etching” [31]) typically involving a 3‐ or 5‐s application of phosphoric acid, compared with manufacturer's recommendations (15 s) may improve bonding effectiveness for specific adhesive systems, aiming to preserve residual hydroxyapatite crystals within the intrafibrillar collagen spaces and reduce the risk of over‐demineralization, thereby potentially improving the long‐term durability of the bonded interface [26, 31, 39]. However, to the best of the authors' knowledge, clinical studies comparing different dentin etching times under clinically relevant conditions are still lacking [40].

Thus, the objective of this study was to evaluate the effect of reduced dentin etching time (5 s) in comparison with manufacturer's recommendations (15 s) on the clinical performance of two universal adhesive systems in noncarious cervical lesions (NCCLs). The following null hypotheses were tested: (a) there is no significant difference in the primary outcome (fracture of material and retention rate) between restorations performed using universal adhesive systems applied on dentin etched for 5 (experimental time) or 15 s (control time); (b) there is no significant difference in the secondary outcomes (marginal staining, marginal adaptation, postoperative sensitivity, and recurrence of caries) between restorations placed using universal adhesive systems applied on dentin etched for 5 (experimental time) or 15 s (control time).

## 2 Methods

### 2.1 Ethics Approval and Protocol Registration

The local research committee approved the study protocol (protocol CPI‐ODO2024FN09). In addition, the institutional research ethics committee reviewed and approved the study protocol and informed consent form (protocol CEC2024055). Written informed consent was obtained from all participants prior to treatment initiation. The experimental design followed the Consolidated Standards of Reporting Trials (CONSORT) statements [41, 42, 43].

### 2.2 Trial Design, Settings, and Location of Data Collection

This randomized, double‐blind (participant and examiner), split‐mouth controlled clinical trial was registered in the Registry of Clinical Trials by the number NCT06465849. This study was conducted at the Universidad de los Andes Health Center (CESA) between November 2023 to January 2024. Participants were informed about the nature and objectives of the study, but they were unaware of which tooth received each specific treatment under evaluation.

### 2.3 Participant Recruitment

Participants were recruited while seeking treatment at the dental clinics of the university. Those who met the eligibility criteria were enrolled consecutively as they attended the screening session, thus constituting a convenient sample. No advertisements were used for participant recruitment.

### 2.4 Sample Size Calculation

The primary outcome of this study was fracture of material/retention rate. Sample size calculation was performed using freely available software (http://www.sealedenvelope.com), based on the fracture of material/retention rate of Scotchbond Universal adhesive, a predecessor of Scotchbond Universal Plus adhesive (Solventum, St Paul, MN, USA), which showed a retention rate of 94% at the 18‐month follow‐up [12]. A minimum sample size of 35 restorations per group was required to detect a difference of 25% among the tested groups, with an *α* of 0.05 and a power of 80%.

### 2.5 Eligibility Criteria

A total of 43 participants were examined by two previously calibrated undergraduate students to ensure that they met the inclusion and exclusion criteria. Clinical examinations were performed using a mouth mirror, explorer, and periodontal probe. All participants received oral hygiene instructions prior to operative treatment.

Within the inclusion criteria, participants had to be in good general health, be older than 18 years, have an acceptable level of oral hygiene according to the Simplified Oral Hygiene Index, and present at least 20 teeth under occlusion. In addition, participants had to have at least four similar NCCLs in four different teeth that required restoration. These lesions had to be noncarious, nonretentive, deeper than 1 mm, involve both the enamel and dentin, be located in vital teeth without mobility, and present cavosurface margin involving no more than 50% of enamel. Within the exclusion criteria, participants with poor oral hygiene (OHI‐S > 3), severe or chronic periodontitis (probing depth > 4 mm with bleeding on probing and clinical attachment loss > 3 mm in more than four teeth), severe bruxism (severe masticatory muscle pain, temporomandibular joint pain, or extreme tooth wear), and use of orthodontic devices or removable prosthesis were excluded from the study.

### 2.6 Randomization Sequence Generation and Allocation Concealment

Randomization process was performed using freely available software (http://www.sealedenvelope.com) by a researcher uninvolved in any experimental phase. Group allocation was recorded on cards contained in sequentially numbered, opaque, sealed envelopes. Allocation assignment was revealed immediately before the restorative procedure by opening the envelope, ensuring concealment of the randomization sequence and minimizing selection bias. Participants and examiners were blinded to group assignment.

### 2.7 Baseline Characteristics of the Selected Teeth

The same two trained and calibrated undergraduate students who were involved in the selection of participants also performed the restorative procedures. The features of the NCCLs were evaluated before the restoration's placement. The degree of dentin sclerosis was evaluated according to an earlier modified scoring system (Table 1) [44]. Lesion dimensions (height, width, and depth in millimeters) and lesion geometry (evaluated by photographs and labeled at < 45°, 45°–90°, 90°–135°, > 135°) were recorded. Other features such as the presence of attrition facets were also recorded. Preoperative (hyper)sensitivity was evaluated prior to treatment by evaluating spontaneous sensitivity, applying an air stream for 10 s from a dental syringe placed 2 cm from the tooth surface, and tactile stimulation using a dental explorer (Table 2).

**TABLE 1:** Dentin sclerosis scale^a^.

Category	Criteria
1	No sclerosis present; dentin is light yellowish or whitish, with little discoloration; dentin is opaque, with little translucency or transparency.
2	More sclerosis than in Category 1 but less than halfway between Categories 1 and 4.
3	Less sclerosis than in Category 4 but more than halfway between Categories 1 and 4.
4	Significant sclerosis present; dentin is dark yellow or even discolored (brownish); glassy appearance, with significant translucency or transparency evident.

^a^ Adapted from Swift et al. [44].

**TABLE 2:** Characteristics of the research participants and the noncarious cervical lesions (NCCLs) per group.

Characteristics of research participants	No. of participants
Sex distribution	
Male	17
Female	19
Age distribution (years)	
20–29	0
30–39	2
40–49	12
> 49	22

Characteristics of NCCL lesions	Number of lesions			
	SUP5	SUP15	GBU5	GBU15
Shape, degree of angle				
< 45	4	2	4	6
45–90	13	17	15	16
90–135	17	15	17	10
> 135	2	2	0	4
Cervico‐incisal height, mm				
< 1.5	6	7	9	5
1.5–2.5	15	11	12	10
2.5–4.0	12	13	13	18
> 4.0	3	5	2	3
Degree of sclerotic dentin				
1	18	10	17	9
2	9	17	14	14
3	9	9	3	10
4	0	0	2	3
Presence of antagonist				
Yes	35	35	34	35
No	1	1	2	1
Attrition facet				
Yes	28	31	31	29
No	8	5	5	7
Preoperative sensitivity (spontaneous)				
Yes	18	12	14	13
No	18	24	22	23
Preoperative sensitivity (air dry)				
Yes	23	19	17	17
No	13	17	19	19
Preoperative sensitivity (touch)				
Yes	11	5	10	9
No	25	31	26	27
Tooth distribution				
Anterior				
Incisor	1	4	2	5
Canines	4	7	9	4
Posterior				
Premolar	27	16	18	19
Molar	4	9	7	8
Arc distribution				
Maxillary	20	12	17	17
Mandibular	16	24	19	19

Abbreviations: GBU15, GBU applied on dentin etched for 15 s; GBU5, Gluma Bond Universal adhesive applied on dentin etched for 5 s; SUP15, SUP applied on dentin etched for 15 s; SUP5, Scotchbond Universal Plus adhesive applied on dentin etched for 5 s.

For operator calibration, the study director initially placed one restoration per group to standardize all protocol steps. Then, two operators (undergraduate students) placed four restorations per group under supervision. Any discrepancies in the restorative protocol were identified and discussed before study initiation. Restorations performed by the operators in the calibration period were not part of the final results of the study. The calibrated operators restored all teeth under the supervision of the study director.

### 2.8 Interventions: Restorative Procedure

A detailed protocol standardizing the interventions is briefly summarized as follows. Preliminary dental prophylaxis of the tooth surface was performed using pumice and water in a rubber cup, followed by rinsing and drying. Shade selection was determined using a shade guide (Vita Classical; Vita Zahnfabrik, Bad Säckingen, Germany). Local anesthesia was administered with 3% mepivacaine (Isocaine, Novocol, Ontario, Canada), and all restorations were placed under rubber dam isolation. No additional mechanical retention or beveling was performed to preserve tooth structure and ensure minimally invasive cavity design.

For all groups, a 35% phosphoric acid (Condac 37; FGM Dental Products, Joinville, SC, Brazil) was applied for 15 s in enamel, and 5 s or 15 s in dentin, according to the tested groups. Then, cavities were rinsed for 10 s. Excess moisture was removed by thoroughly air‐drying to maintain a moist dentin surface without pooling. Absorbent papers were not used.

Then, Scotchbond Universal Plus adhesive (SUP; batch# 8321939; Solventum, St Paul, MN, USA, also known as Single Bond Universal Plus in some countries) and Gluma Bond Universal (GBU; batch# K010046; Heraeus Kulzer, Hanau, Germany; also known as IBond Universal in some countries) were applied according to the following groups: (1) SUP5: Scotchbond Universal Plus adhesive (ultramild universal adhesive) [45, 46, 47, 48, 49] applied on dentin etched for 5 s (experimental group); (2) SUP15: Scotchbond Universal Plus adhesive applied on dentin etched for 15 s (control group); (3) GBU5: Gluma Bond Universal adhesive (intermediate strong universal adhesive) [48, 49, 50, 51, 52] applied on dentin etched for 5 s (experimental group); (4) GBU15: Gluma Bond Universal adhesive applied on dentin etched for 15 s (control group). Adhesives were applied to the etched enamel and dentin for approximately 20 s, gentle air for 5 s, and finally light‐curing for 10 s at 1000 mW/cm^2^ (SmartLite Focus; Dentsply Sirona, DeTrey, Konstanz, Germany) according to the manufacturer's recommendations (Table 3).

**TABLE 3:** Application mode of the adhesive system and composite resin in the different groups.

Materials	Composition	Application mode only in the etch‐and‐rinse mode
Condac 37 (FGM Dental Group, Joinville, SC, Brazil) [010823]	37% phosphoric acid	Apply phosphoric acid for 15 s in enamel. Apply etchant on dentin for 5 or 15 s^a^ (control). Wash the surface with plenty of water for 10 s and dry the cavity so that the dentin does not get dehydrated, but without the accumulation of water on the surface. Apply the adhesive to the entire preparation with a microbrush and rub it in for 20 s. If necessary, rewet the disposable applicator during treatment. Direct a gentle stream of air over the liquid for about 5 s until it no longer moves and the solvent has evaporated completely. Light cure for 10 s at 1000 mW/cm^2^.
Scotchbond Universal Plus Adhesive (3 M Oral Care, St Paul, MN, USA) [8321939] pH ≈2.5–2.7 (ultra‐mild)	10‐MDP, 1,3‐benzenediol 2‐(2‐hydroxyethoxy)ethyl 3‐hydroxypropyl diethers, HEMA, 2‐Propenoic acid, 2‐methyl‐, 3‐(triethoxysilyl)propyl ester, reaction products with silica, silanes (γ‐MPTES/APTES), ethanol, water, CQ, a copolymer of acrylic and itaconic acid, copper (II) acetate monohydrate	
Gluma Bond Universal (Heraeus Kulzer, Hanau, Germany) [K010046] pH ≈1.5–1.8 (intermediate strong)	10‐MDP, 4‐META, Bis‐GMA, 4‐ethyl dimethyl aminobenzoate, cetylamine hydrofluoride, initiator, acetone (25%–50%), water.	
Vittra APS (FGM Dental Group, Joinville, SC, Brazil) [130720]	UDMA, TEGDMA, photoinitiator composition (Advanced Polymerization System), coinitiators, stabilizer, and silane; 200‐nm spheroidal zircon‐silicate fillers, wt/vol 72%–82%/52%–60%	Placed in increments of 2 mm. Light‐curing for 20 s each layer at 1000 mW/cm^2^.

Abbreviations: 10‐MDP, methacryloyloxydecyl dihydrogen phosphate; 4‐META, 4‐methacryloxyethyl trimellitate anhydride; APTES, 3‐(aminopropyl)triethoxysilane; Bis‐GMA, bisphenol A‐glycidyl methacrylate; HEMA, 2‐hydroxyethyl methacrylate; TEGDMA, triethylene glycol dimethacrylate; UDMA, urethane dimethacrylate; γ‐MPTES, γ‐methacryloxypropyltriethoxysilane.

^a^ Manufacturer's recommendation.

The operators restored the cervical tooth anatomy by applying three increments of resin composite (Vittra APS, FGM Dental Products, Joinville, SC, Brazil). Each increment was light‐cured for 10 s at 1000 mW/cm^2^ (SmartLite Focus; Dentsply Sirona, DeTrey, Konstanz, Germany). The restorations were finished immediately with fine diamond burs (#862F.FG.010 and #862EF.FG.010; Jota AG, Rüthi, Switzerland) and polished with coarse, medium and fine polishing points (Jiffy; Ultradent, South Jordan, UT, USA) (Figure 1).

**FIGURE 1:**
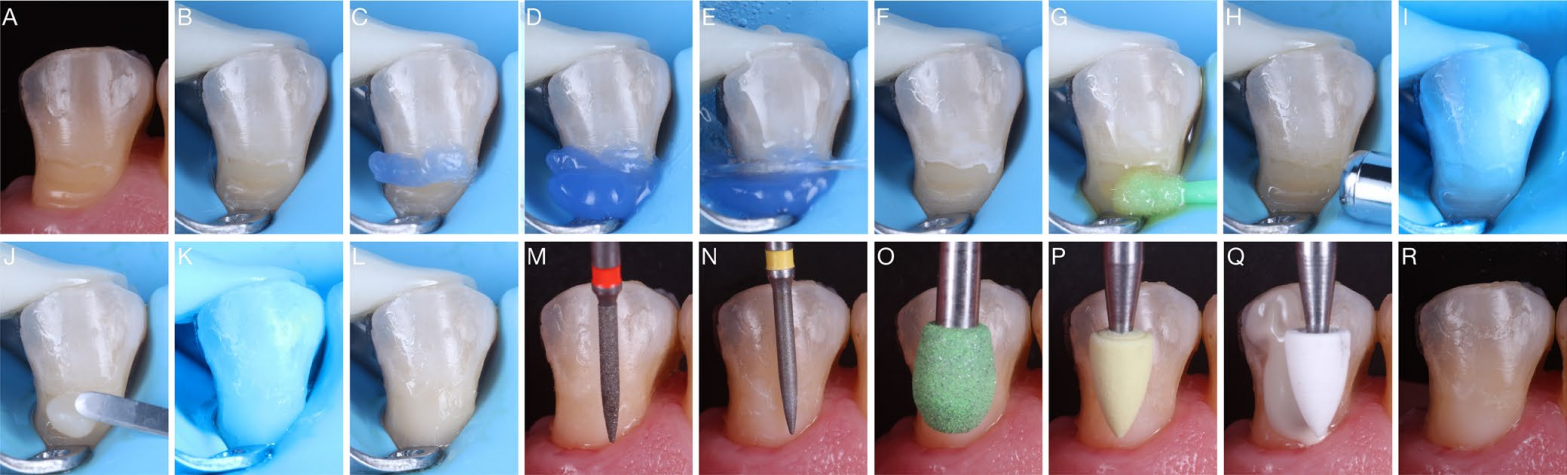
(A) Initial condition; (B) rubber dam isolation; (C) enamel etching; (D) dentin etching for 5 or 15 s, according to the experimental group; (E) rinsing for 10 s; (F) air drying while maintaining dentin moisture; (G) adhesive application; (H) solvent evaporation for 5 s; (I) light cure of the adhesive for 10 s; (J) incremental application of resin composite; (K) light curing of the resin composite for 20 s; (L) restoration prior to the finishing and polishing; (M) finishing with a fine diamond bur; (N) finishing with an ultrafine diamond bur; (O) polishing with a coarse‐grit (green) polishing point; (P) polishing with a medium‐grit (yellow) polishing point; (Q) polishing with fine‐grit (white) polishing point and polishing paste; (R) final condition.

### 2.9 Clinical Evaluation

Two experienced, blinded, and calibrated examiners, not involved in the restorative procedures performed the clinical evaluation. Inter‐ and interexaminer agreement of at least 85% was necessary before beginning the evaluation.

All parameters during the evaluation were recorded using a standardized paper case report form. The evaluation paper had to be sent to the research staff after each observation so that the examiners were blinded to the group assignments during follow‐up recalls. The updated version of the FDI was used immediately after the restorative procedure (baseline) and after 6 months of clinical service [53, 54]. For that criteria, only clinically relevant measures of the performance of the adhesives were evaluated (Table 4). The primary outcome was fracture of material and retention (F1, functional properties). However, the following secondary outcomes were also evaluated: functional properties (F2. Marginal adaptation), biological (B1. Caries lesions at restoration margins; B2. Dental Hard tissues defects at the restoration margin; B3. Postoperative hypersensitivity and pulpal status) and esthetic (A2. Marginal staining) properties.

**TABLE 4:** Updated World Dental Federation (FDI) criteria used for clinical evaluation [53, 54].

	Functional properties^a^		Biological properties			Esthetic property
	F1. Fracture of material and retention	F2. Marginal adaptation	B1. Caries lesions at restoration margins	B2. Dental hard tissue defects at the restoration margin	B3. Postoperative (hyper‐) sensitivity	A2. Marginal staining
1. Clinically very good (sufficient)	Restoration is completely present without deficiencies detectable after air drying. No crack, chipping/delamination, or material bulk fracture	Harmonious outline, no gaps detectable by gentle probing, using a *250‐μm probe*	No caries/demineralization at the restoration mar gin detectable after air drying.	Visual examination	No hypersensitivity.	No marginal staining detectable after air drying.
2. Clinically good (sufficient)	Restoration is completely present with minor deficiencies detectable after air drying, e.g., insignificant material chippin or one hairline crack.	Slight deficiencies of marginal adaptation after air drying. Minor, superficial marginal gap(s) or ditching.	First visible signs of a noncavitated caries lesion at the restoration margin detectable after air drying.	Intact dental hard tissue without crack lines and fractures at the restoration margin	Low hypersensitivity for a limited period of time	Minor marginal staining detectable after air drying
3. Clinically sufficient/satisfactory (sufficient)	Restoration is present with deficiencies detectable without air drying, e.g., hairline cracks or distinct material loss (chipping). Material loss can mainly be corrected by refurbishment if needed	Distinct deficiencies of marginal adaptation without air drying: marginal gap(s) or ditching (width < 250 μm and/or depth < 2 mm).	Established, noncavitated caries lesion or microcavity at the restoration margin detectable without air drying.	Minor vertical/horizontal hairline crack lines in enamel at the restoration margin	Patient reports distinct postoperative hypersensitivity or distinct pain on chewing and/or cold/warm food items reported by the patient for a prolonged period of time (> 1 week). Normal (short) or more intense reaction to sensitivity test on cold.	Distinct marginal staining detectable without air drying but not displeasing. Refurbishment is possible.
4. Clinically unsatisfactory (partially insufficient)	Chipping fractures which damage marginal quality; bulk fractures with or without partial loss (less than half of the restoration).	Localized but severe deficiencies of marginal adaptation: width ≥ 250 μm and/or depth ≥ 2 mm marginal gap(s). Partially loose/lost restoration. Repair is possible.	Localized dentin cavity (width > 250 μm, depth > 2 mm) at the restoration margin. Repair is possible.	Distinct enamel chipping or enamel fracture at the restoration margin. If necessary, deficiencies can be corrected by refurbishment	Patient reports severe/persistent, postoperative hypersensitivity or persistent pain on chewing and/or cold/warm food items reported by the patient for a prolonged period of time (> 1 month) AND/OR intense reaction to sensitivity test on cold. Both symptoms indicate irreversible pulpitis. Endodontic treatment requires access cavity only.	Localized, displeasing deep marginal staining. Marginal staining can be removed/improved by repair.
5. Clinically poor (entirely insufficient)	(Partial or complete) loss of restoration. Repair not possible/reasonable.	Generalized and severely compromised marginal adaptation: width ≥ 250 μm and/or depth ≥ 2 mm. Complete loose/lost restoration. Repair not possible/reasonable.	Extensive dentin cavity at the restoration margin. Repair not possible/reasonable.	Severe marginal (enamel) fracture, partially fractured cusp or ridge at the restoration margin. Repair is possible	Irreversible pulpitis, nonvital tooth, pulp necrosis with or without periapical periodontitis after restoration placement. Endodontic treatment requires replacement of the restoration.	Generalized, displeasing deep marginal staining. Repair not possible/reasonable.

^a^ Part referent to indirect restoration was removed from the description.

The postoperative sensitivity was performed 1 week after the restorative procedure by applying an air stream from a dental syringe for 10 s at 2 cm from the tooth surface. Also, one criterion called miscellaneous was evaluated: participant's view (M1) [53, 54]. After 7 days of the restorative procedure, participants filled out a 5‐point Likert scale to measure satisfaction from the participant's perspective. The scale was structured as follows: (1) Very satisfied; (2) Satisfied; (3) Neither satisfied nor dissatisfied; (4) Dissatisfied; and (5) Very dissatisfied. Participants selected the option that best reflected their level of satisfaction with the procedure. In cases of dissatisfaction, a detailed report was provided on aspects such as pain, hypersensitivity, chewing comfort, cleanability, contours, or esthetics. Examiners evaluated all the restorations and gave their scores individually. If any disagreement occurred, examiners had to reach a consensus before the participant left.

These variables were ranked according to the following criteria: updated version of FDI criteria (clinically very good [VG], clinically good [GO], clinically sufficient/satisfactory [SS], clinically unsatisfactory [UN], and clinically poor [PO]) [53, 54]. Both examiners evaluated all the restorations once and independently. In case of disagreement during the evaluations, a consensus had to be reached before the participant was dismissed. All restorations scored as clinically unsatisfactory or poor according to the FDI criteria at one recall were considered cumulative failures at the next follow‐up evaluation. Each failed restoration owing to retention loss was replaced with a new composite resin restoration. These new restorations were not included in the present study for further evaluation.

### 2.10 Statistical Analysis

The statistician was blinded to the study group types, and the statistical analysis followed the intention‐to‐treat protocol according to CONSORT's suggestion [42]. Descriptive statistics were used to summarize clinical outcomes. The Kolmogorov–Smirnov test assessed data normality. Due to nonnormal distribution, risk ratios and relative risks of experimental approaches (5 s) relative to the control approach (15 s), as well as SUP versus GBU were also calculated with 95% confidence intervals (CIs).

For secondary outcomes (marginal adaptation, caries lesions at restoration margins, dental hard tissues defects at the restoration margin, postoperative hypersensitivity and pulpal status and marginal staining) in each overall FDI parameter, the differences between the four groups' ratings after 6 months were tested using the Kruskal–Wallis analysis of variance rank and Mann–Whitney *U* test (*α* = 0.05). The relative risk was calculated relative to the control group. The 95% CI was also reported. Cohen's *κ* statistic was used to test interexaminer agreement (*α* = 0.05). All analyses were performed using Statistica for Windows (version 7.0, StatSoft Inc., Tulsa, OK, USA).

## 3 Results

In the total of 43 participants evaluated, 7 were excluded and 36 were recruited after accepting the terms of the research (Figure 2). A total of 144 restorations were placed, in 36 participants (17 male; mean age 52.9 [37–71] and 19 females; mean age 55.1 [38–74]) with 36 restorations allocated to each experimental group (Figure 2). All restorative procedures were implemented exactly as planned, and no protocol modifications occurred.

**FIGURE 2:**
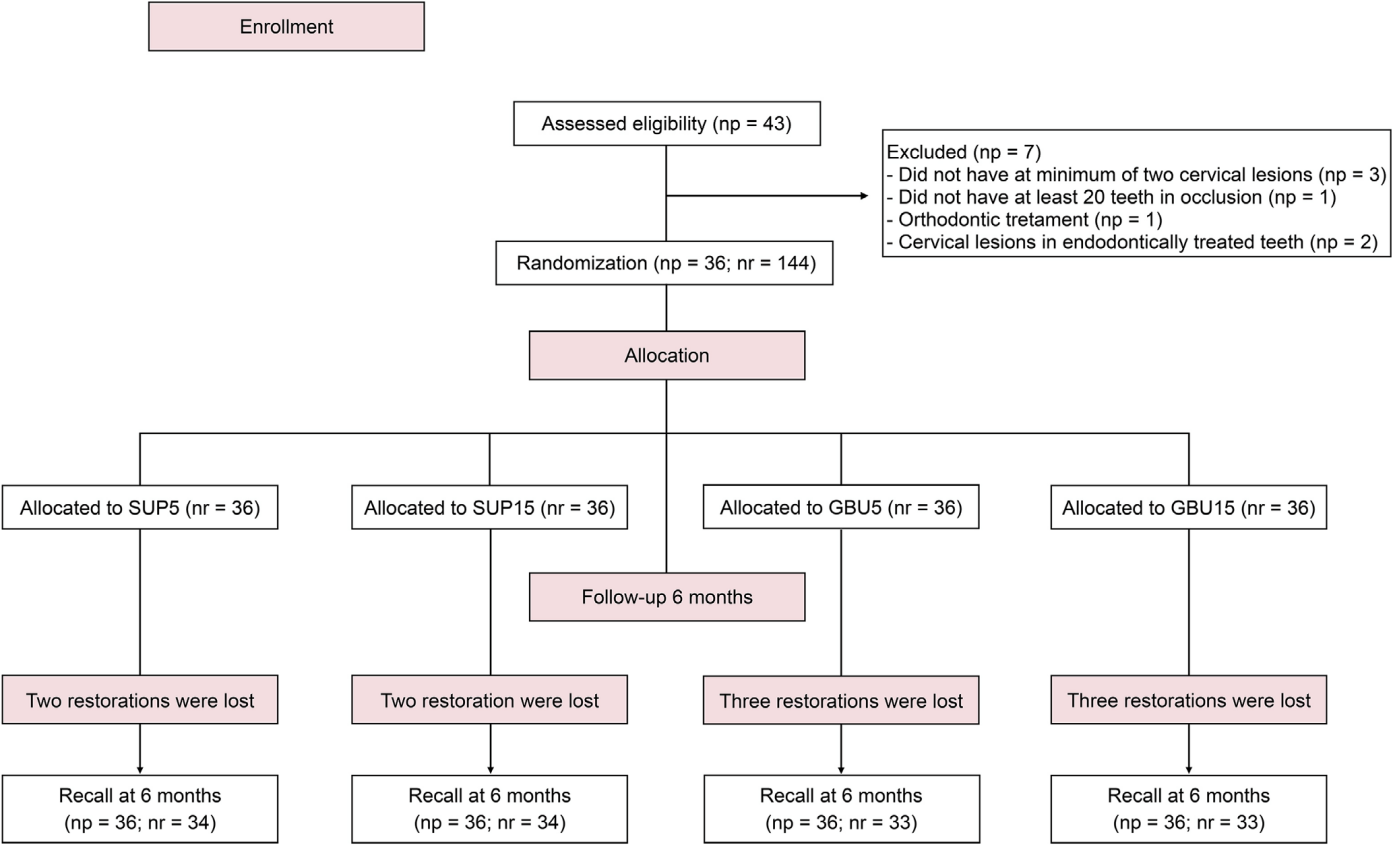
Participant flow diagram illustrating the different phases of the study design. Np, number of participants; Nr, number of restorations; SUP5: Scotchbond Universal Plus adhesive applied to dentin etched for 5 s; SUP15: Scotchbond Universal Plus adhesive applied to dentin etched for 15 s; GBU5: Gluma Bond Universal adhesive applied to dentin etched for 5 s; GBU15: Gluma Bond Universal adhesive applied to dentin etched for 15 s.

Baseline demographic data of the participants and the characteristics of the restored lesions are presented in Table 2. The overall Cohen's *κ* statistics showed almost perfect agreement among the examiners at the 6‐month (0.87). All participants were available for evaluation at baseline and at the 6‐month recall, resulting in a 100% recall rate. Only outcome parameters in which at least one restoration exhibited a deviation from the ideal score are reported below.

### 3.1 Functional Properties: Fracture of Material and Retention (F1)

Follow‐up data for fracture of material and retention are presented in Table 5. After 6 months of clinical evaluation, 10 restorations were lost (two for SUP5, two for SUP15, three for GBU5, and three for GBU15).

**TABLE 5:** Number of evaluated restorations for each experimental group classified according to the World Dental Federation (FDI).

FDI criteria		Baseline				6 months			
		SUP5	SUP15	GBU5	GBU15	SUP5	SUP15	GBU5	GBU15
F1. Fracture of material and retention	VG	36	36	36	36	33	34	32	32
	GO	—	—	—	—	—	—	—	—
	SS	—	—	—	—	1	—	1	1
	UN	—	—	—	—	—	—	—	—
	PO	—	—	—	—	2	2	3	3
F2. Marginal adaptation	VG	36	36	36	36	29	30	24	26
	GO	—	—	—	—	3	4	9	5
	SS	—	—	—	—	2	—	—	2
	UN	—	—	—	—	—	—	—	—
	PO	—	—	—	—	—	—	—	—
B1. Caries lesions at restoration margins	VG	36	36	36	36	34	34	33	33
	GO	—	—	—	—	—	—	—	—
	SS	—	—	—	—	—	—	—	—
	UN	—	—	—	—	—	—	—	—
	PO	—	—	—	—	—	—	—	—
B2. Dental hard tissue defects at the restoration margin	VG	36	36	36	36	34	34	33	33
	GO	—	—	—	—	—	—	—	—
	SS	—	—	—	—	—	—	—	—
	UN	—	—	—	—	—	—	—	—
	PO	—	—	—	—	—	—	—	—
B3. Postoperative (hyper‐) sensitivity	VG	36	36	36	36	34	34	32	32
	GO	—	—	—	—	—	—	—	—
	SS	—	—	—	—	—	—	—	—
	UN	—	—	—	—	—	—	—	—
	PO	—	—	—	—	—	—	—	—
A2. Marginal staining	VG	36	36	36	36	34	34	32	32
	GO	—	—	—	—	—	—	—	—
	SS	—	—	—	—	—	—	—	—
	UN	—	—	—	—	—	—	—	—
	PO	—	—	—	—	—	—	—	—

*Note:* SUP5 (Scotchbond Universal Plus adhesive applied on dentin etched for 5 s); SUP15 (SUP applied on dentin etched for 15 s); GBU5 (Gluma Bond Universal adhesive applied on dentin etched for 5 s); GBU15 (GBU applied on dentin etched for 15 s).

Abbreviations: GO: clinically good; PO: clinically poor; SS: clinically sufficient/satisfactory; UN: clinically unsatisfactory; VG: clinically very good.

According to updated FDI criteria, the 6‐month retention/fracture rates (95% CI) were 97.2% (87.3–100) for SUP‐5, 97.2% (87.3–100) for SUP15, 94.4% (83.1–100) for GBU5, and 94.4% (83.1–100) for GBU15 (Table 5). No statistically significant differences were detected among any of the experimental groups at the 6‐month follow‐up (*p* > 0.08; Table 6).

**TABLE 6:** Absolute risk (95% CI) and relative risk (95% CI) for outcome fracture of material and retention for different groups after 6 months of clinical evaluation.

	Absolute risk (95% CI)	Pairwise comparison	Relative risk (95% CI)
SUP15	5.5 (1.5–18.1)	SUP15 vs. SUP5	1.0 (1.4–67.1)^a^
SUP5	5.5 (1.5–18.1)	SUP5 vs. GBU5	1.5 (2.6–84.4)^b^
GBU15	8.3 (2.8–21.8)	GBU15 vs. GBU5	1.0 (2.1–46.2)^a^
GBU5	8.3 (2.8–21.8)	SUP15 vs. GBU15	1.5 (2.1–84.4)^b^

^a^ Comparisons were performed for each adhesive in relation to their control (SUP15 and GBU15).

^b^ Comparisons were performed for each time point in relation to the adhesive.

### 3.2 Functional Properties: Marginal Adaptation (F2)

According to the updated FDI criteria, 25 restorations (five for SUP5, four for SUP15, nine for GBU5 and seven for GBU15) presented minimal marginal adaptation defects (Table 5). No statistically significant differences were detected among the groups at the 6‐month follow‐up (*p* > 0.05; Table 5).

### 3.3 Other Parameters (B1–B3)

After 6 months evaluation, two restorations showed minor postoperative (hyper‐) sensitivity. No restoration exhibited caries lesions at restoration margins or dental hard tissues defects at the restoration margin (B1–B3, biological properties) immediately after the restorative procedures or at the 6‐month follow‐up according to the updated FDI criteria (Table 5).

### 3.4 Esthetic Properties: Marginal Staining (A2)

After 6 months evaluation, two restorations (one for GBU5, one for GBU15) showed minor marginal staining according to the updated FDI criteria (Table 5). No statistically significant differences were detected among the groups at the 6‐month follow‐up (*p* > 0.05; Table 5).

### 3.5 Miscellaneous Parameter

Despite the occurrence of restoration loss in some groups, all participants reported being very satisfied with their restorations according to the participant's view criterion (M1) at the 6‐month follow‐up [53, 54].

## 4 Discussion

Universal adhesive systems represent a recent advance in dental bonding technology and are formulated as single‐component materials intended to enhance clinical flexibility [8]. Owing to this multifunctional design, universal adhesives can be effectively employed in a broad spectrum of clinical situations due to their versatility and ease of use for clinicians. In this context, they may be applied using different protocols, including the etch‐and‐rinse approach, analogous to a two‐step etch‐and‐rinse system, which relies on phosphoric acid conditioning of both enamel and dentin; the self‐etch strategy, similar to a one‐step self‐etch system, which streamlines the procedure by omitting a separate etching step; and selective enamel etching, which integrates key features of both techniques [6].

Current laboratory studies indicate that, under in vitro conditions, universal adhesives often demonstrate superior immediate [19, 20, 21] and long‐term performance [19, 22, 23], when applied using the self‐etch strategy compared with the etch‐and‐rinse approach. Furthermore, a recent systematic review of in vitro studies suggested that, from the perspective of long‐term bonding performance or procedural simplification, the self‐etch mode may be preferred [24]. However, for the universal adhesives evaluated in the present randomized clinical trial, this assumption appears, at minimum, controversial.

For Scotchbond Universal, the predecessor of Scotchbond Universal Plus used in this study, several in vitro studies have reported that the etch‐and‐rinse application mode results in higher bond strength and reduced gap formation compared with the self‐etch strategy [28, 29]. Similarly, a recent in vitro study showed that for Scotchbond Universal Plus, an ultra‐mild universal adhesive (pH ≈2.5–2.7), the etch‐and‐rinse technique resulted in superior bond strength compared to the self‐etch application [39]. Moreover, for Gluma Bond Universal, an intermediate‐strong universal adhesive (pH ≈1.5–1.8), in vitro studies have likewise shown improved bonding performance when applied using the etch‐and‐rinse approach rather than the self‐etch method [28, 30]. These findings are consistent with a recent systematic review indicating that, for universal adhesives classified as ultra‐mild and intermediate‐strong, the etch‐and‐rinse technique demonstrated superior immediate and long‐term bonding performance compared with the self‐etch approach [36].

Corroborating these laboratory findings, clinical trials have reported that the long‐term performance of Scotchbond Universal [46, 47] and Gluma Bond Universal [52] in etch‐and‐rinse mode may be slightly superior to that observed with the self‐etch mode. These outcomes raise concerns regarding the hybridization efficiency of universal adhesives when used in self‐etch mode under clinical conditions characterized by denser smear layers, such as those found in NCCLs. This underscores the importance of randomized clinical trials to evaluate both the short‐ and long‐term performance of universal adhesives and their application strategies.

Nevertheless, dentin acid etching is associated with specific challenges. One major concern is the formation of an extensive demineralization pattern, characterized by an increased depth of demineralized zone and a higher density of exposed dentinal tubules [26]. Consequently, discrepancies may arise between the depth of demineralization and the extent of adhesive monomer infiltration, leaving zones of exposed collagen beneath the hybrid layer [32]. These regions are particularly susceptible to degradation over time due to the activation and release of endogenous matrix metalloproteinases and cysteine cathepsins [33], which are triggered by phosphoric acid and acidic monomers present in adhesive systems [55].

A second limitation associated with acid etching of dentin is the removal of hydroxyapatite surrounding collagen fibers, resulting in a reduction of available calcium ions. The adhesive performance of the functional monomer 10‐MDP is primarily attributed to its ability to establish stable chemical bonds with mineralized dental tissues via calcium‐mediated interactions [3, 11]. These interactions result in the formation of highly ordered, water‐insoluble calcium–phosphate nanolayers at the adhesive–hydroxyapatite interface [16].

Although the extent of nanolayer formation is influenced by the effective concentration of 10‐MDP applied to dentin [17], the presence of residual hydroxyapatite at the adhesive interface is essential to achieve meaningful chemical bonding. When such interactions are preserved, the resulting tooth–restoration interface is better able to withstand functional loading and hydrolytic degradation.

Conversely, excessive removal of hydroxyapatite may compromise interfacial chemistry by limiting the availability of calcium ions, thereby favoring weaker and less durable ionic interactions. Since the demineralizing capacity of phosphoric acid is influenced by etching duration [26, 31], a straightforward alternative to maintain higher calcium content within the hybrid layer is to reduce the dentin etching time.

Although manufacturers typically recommend a dentin etching time of 15 s, accumulating experimental evidence indicates that this duration is sufficient to cause extensive mineral depletion and pronounced collagen exposure [31], suggesting that it may be excessive for establishing an optimal dentin etching pattern [26, 31]. In this context, reducing dentin etching time appears to be a practical strategy that does not introduce additional clinical steps, thereby preserving the simplicity and reduced technique sensitivity that are among the main advantages of universal adhesives. However, to date, clinical studies evaluating different dentin etching times using universal adhesives in NCCLs are lacking [40].

In this context, the current study evaluated the effect of reduced dentin etching time (5 s) on the clinical performance of universal adhesives compared with the conventional 15‐s etching time commonly recommended by manufacturers. Although several in vitro studies have demonstrated that shorter dentin etching times (3 or 5 s) may improve bonding effectiveness for specific adhesive systems [26, 31, 39], to the best of the authors' knowledge, this is the first randomized clinical trial to evaluate the impact of reduced dentin etching time on the performance of two universal adhesive systems with different solvent types in NCCLs.

In this regard, the clinical performance of adhesive systems is commonly assessed through clinical trials in NCCLs, as these lesions are particularly challenging due to the lack of macromechanical retention, the predominance of dentin substrate, and the limited enamel surface. Accordingly, in the present study, clinical performance was primarily evaluated based on restoration retention/material fracture, while secondary outcomes included marginal staining, marginal adaptation, postoperative sensitivity, and recurrence of caries.

In this study, two universal adhesive systems with different pH values were evaluated: Scotchbond Universal Plus, with a pH ≈2.5–2.7 (ultra‐mild), and Gluma Bond Universal, with a pH ≈1.5–1.8 (intermediate strong). Regardless of adhesive pH, reducing the dentin etching time to 5 s did not result in significant differences in clinical performance compared with the conventional 15‐s protocol. Furthermore, after 6 months of clinical service, retention rates were comparable between groups, reaching 97.2% (95% CI; 87.3–100) for Scotchbond Universal Plus and 94.4% (95% CI; 83.1–100) for Gluma Bond Universal, in agreement with findings from recent clinical studies that evaluated these adhesives [48, 51, 52].

These results contrast with those reported in recent in vitro studies [26, 39], in which reduced dentin etching time was associated with improved bond strength compared with the manufacturer‐recommended etching time, both immediately and after aging (6 months or 4 years), for Scotchbond Universal Plus and its predecessor (Scotchbond Universal). On the other hand, no in vitro data are currently available comparing different dentin etching times for Gluma Bond Universal; however, a recent clinical study [49] shows that, after 6 months, the retention rate of Gluma Bond Universal was lower than in the present study. This reinforces the idea that, for some adhesives, acid etching of dentin yields better results than the self‐etch technique, even when etching is for a short time. The present clinical findings suggest that a shorter etching time does not compromise smear layer removal or dentin hybridization, while potentially reducing procedural and chair time.

Additionally, no differences in retention rates were observed between the two universal adhesives, despite differences in their acidity. This finding contrasts with a previous systematic review indicating that adhesive pH influences the immediate and long‐term bonding performance of universal adhesives. Nevertheless, such laboratory‐based conclusions should be interpreted with caution due to the heterogeneity among universal adhesives and the well‐recognized limitations of in vitro studies, which may not accurately reflect clinical performance.

Moreover, after 6 months, marginal adaptation rates were 85.3% (95% CI; 69.9–93.6) for SUP5, 88.2% (95% CI; 73.4–95.3) for SUP15, 72.7% (95% CI; 55.8–84.9) for GBU5, and 78.8% (95% CI; 62.2–89.3) for GBU15, with no statistically significant differences among the groups. In addition, regarding the other secondary outcomes, no significant differences were observed between reduced (5 s) and conventional (15 s) dentin etching times for either adhesive. Similarly, no significant differences were observed between the two universal adhesives, which is consistent with recent clinical studies comparing these adhesives [48, 51, 52]. Thus, none of the null hypotheses tested in this study were rejected.

Finally, this study has some limitations. First, the sample size may limit the detection of small but clinically relevant differences. Second, only one universal adhesive was evaluated within each pH category, precluding generalization of the findings to other formulations. Third, although it was done intentionally to eliminate that variable, the resin corresponds to a different brand than the adhesives tested. Finally, a longer follow‐up period may reveal differences in clinical performance associated with reduced dentin etching time. Therefore, further clinical studies investigating other universal adhesives, with a larger sample size and longer follow‐up periods, are warranted.

## 5 Conclusion

Within the limitations of this current study, it was concluded that a reduced dentin etching time for 5 s did not negatively affect the primary outcome (fracture of material and retention rate) and secondary outcomes (marginal staining, marginal adaptation, postoperative sensitivity, and recurrence of caries) of resin restorations in NCCLs after 6 months, in comparison with dentin etched for 15 s, when universal adhesives were used. Therefore, shortening the dentin etching time represents a viable clinical approach, reducing chair time without compromising restoration performance.

## Author Contributions

**Mario Felipe Gutiérrez:** conceptualization, formal analysis, funding acquisition, investigation, methodology, supervision, writing – original draft preparation, writing – review and edition; **Natalia Amo‐Nicolás:** investigation, methodology, writing – review and editing; **Alondra Beniscelli‐Vásquez:** investigation, methodology, writing – review and editing; **Romina Aliaga‐Gálvez:** methodology, writing – original draft preparation, writing – review and editing; **Alessandro D. Loguercio:** conceptualization, formal analysis, funding acquisition, investigation, methodology, supervision, writing – original draft preparation, writing – review and editing.

## Funding

This project was partially funded by ANID (Agencia Nacional de Investigación y Desarrollo) through Initiation Fondecyt (Fondo Nacional de Desarrollo Científico y Tecnológico—Chile) grant number 11221070 (Chile; M.F.G.). Additionally, this study received partial support from the National Council for Scientific and Technological Development (CNPq) under grant 304444/2025‐1 (Brazil; A.D.L.) and from the Coordenação de Aperfeiçoamento de Pessoal de Nível Superior—Brasil (CAPES) under Finance Code 001.

## Conflicts of Interest

The authors declare no conflicts of interest.

## Data Availability

The data that support the findings of this study are available on request from the corresponding author. The data are not publicly available due to privacy or ethical restrictions.
